# Experiences of Racial Discrimination Among Community-Dwelling Older Adults of Color and Nursing Home Staff of Color

**DOI:** 10.1093/geroni/igaa057.2406

**Published:** 2020-12-16

**Authors:** Manka Nkimbeng, Sarah LaFave, Sarah Szanton

## Abstract

This symposium will present results of three qualitative studies that explored experiences of racial discrimination among older adults and their professional caregivers. Our first study reports on interviews conducted with older community-dwelling African American adults about their perceptions of and experiences with structural racial discrimination. Participants reported exposure that has accumulated over the life course and across contexts, including through limited access to educational and employment opportunities and disproportionate exposure to neighborhood violence and unhealthy products. Our second study reports findings from interviews conducted with older community-dwelling African immigrants about their experiences with acculturation and racial discrimination. Older African immigrants reported several types of discrimination, had developed unique strategies to cope with perceived discrimination and described how it impacted domains of health and wellbeing. Our third study reports on experiences with discrimination among U.S-born and immigrant staff of color caring for residents in high minority proportion nursing homes. Findings indicate that although staff of color are valued for the diversity they contribute to the workforce, they are also exposed to discriminatory events. In addition, tensions exist between U.S. and non-U.S born staff of color. In this symposium, we will discuss differences in experiences between U.S.-born and immigrant participants, levels of discrimination experienced (interpersonal, institutional, structural), and discrimination across contexts. We will also discuss intersectionality of race, ethnicity, immigrant status, language, gender, age, and class in relation to experiences of discrimination. We will reflect on the clinical and policy implications of our findings.

